# Frequency and source of worries in an International sample of pregnant and postpartum women during the Covid-19 pandemic

**DOI:** 10.1186/s12884-021-04241-2

**Published:** 2021-11-12

**Authors:** Diego F. Wyszynski, Sonia Hernandez-Diaz, Vanessa Gordon-Dseagu, Noemi Ramiro, Archana Basu, Hannah H. Kim, Karestan C. Koenen

**Affiliations:** 1 Pregistry, Los Angeles, CA USA; 2 Harvard T.H. Chan School of Public Health, Boston, MA USA; 3 Massachusetts General Hospital, Boston, MA USA

**Keywords:** COVID-19, Pregnancy, Postpartum, Worry, Mental health, survey

## Abstract

**Background:**

Pregnant and postpartum women face unique challenges and concerns during the COVID-19 pandemic. Thus far, no studies have explored the factors associated with increased levels of worry in this population globally. The current study sought to assess the frequency and sources of worry during the COVID-19 pandemic in an international sample of pregnant and postpartum women.

**Methods:**

We conducted an anonymous, online, cross-sectional survey in 64 countries between May and June 2020. The survey was available in 12 languages and hosted on the Pregistry platform for COVID-19 studies. Participants were sought mainly on social media platforms and online parenting forums. The survey included questions related to demographics, level of worry, support, stress, COVID-19 exposure, frequency of media usage, and mental health indicators.

**Results:**

The study included 7561 participants. Eighty-three percent of all participants indicated that they were either ‘somewhat’ or ‘very’ worried. Women 13–28 weeks pregnant were significantly more likely to indicate that they were ‘very worried’ compared to those who were postpartum or at other stages of pregnancy. When compared with women living in Europe, those in Africa, Asia and Pacific, North America and South/Latin America were more likely to have increased levels of worry, as were those who more frequently interacted with social media. Different forms of support and stress also had an impact upon level of worry, while indicators of stress and anxiety were positively associated with worry level.

**Conclusion:**

Pregnant and postpartum women are vulnerable to the changes in societal norms brought about by the COVID-19 pandemic. Understanding the factors associated with levels of worry within this population will enable society to address potential unmet needs and improve the current and future mental health of parents and children.

**Supplementary Information:**

The online version contains supplementary material available at 10.1186/s12884-021-04241-2.

## Background

In December 2019, a novel coronavirus was identified in Wuhan, China [1]. The virus has subsequently been named SARS-CoV-2 and identified as the cause of coronavirus disease (COVID-19) [2]. COVID-19 has spread globally and caused significant disruption to the socio-economic status of countries, regions, and individuals [3, 4], as well as to the provision of healthcare [5, 6]. COVID-19 and the public health control measures required to prevent the spread of the virus have resulted in significant changes to every aspect of life including reduced employment, social isolation, limited travel, and social distancing. Pregnant and recently pregnant women are especially impacted by these changes, which have the potential to adversely affect their wellbeing as well as that of their offspring [7–12]. Furthermore, they are especially vulnerable to the changes and reductions in the provision of health care occurring presently [13, 14].

Efforts to prevent the spread of COVID-19, such as strict lockdowns, have affected the health-seeking behaviors of expecting mothers, for example, by reducing attendance at antenatal appointments [15, 16]. The fear of getting infected has been found to be the main source of increased maternal anxiety [2]. Early mother-baby separation due to compulsory or voluntary quarantine, another important source of maternal anxiety, may also have negative effects on infants’ feeding and early development [17, 18]. Therefore, mother–child bonding is at high risk of being negatively impacted by the COVID-19 pandemic [19].

Previous research has shown that, generally, pregnant and recently pregnant women require additional support from family, friends, health care providers, and the wider community [20] and the importance of such support has been well-documented [21–23]. However, the social distancing required to control COVID-19 has made accessing such support more difficult. Research programs, such as the International Registry of Coronavirus Exposure in Pregnancy (https://ircep.pregistry.com), have been recently established to assess the short- and long-term impact COVID-19 plays on the health and access to care of pregnant and postpartum women and their offspring, including in the lower socio-economic status and black and minority ethnic populations [24, 25].

Restrictions to normal life, reduced employment, social isolation, limited travel, and social distancing are having a deleterious impact on the physical and mental health of individuals. Indicators of physical health, such as amount of regular exercise, weight loss, and healthy eating have declined, while those with chronic medical conditions have seen their health care disrupted [26–29]. Current evidence also reports declining mental health within the general population, and increased incidence of conditions such as anxiety, depression, stress, post-traumatic stress disorder (PTSD), insomnia, and substance abuse as a consequence of the COVID-19 pandemic [26, 30–32].

There is a dearth of data related to the impact of COVID-19 on the levels of worry experienced by pregnant and postpartum women. We conducted an anonymous online international cross-sectional survey which sought to assess the frequency, sources, and influencers of worry in these populations during the COVID-19 pandemic.

## Methods

We conducted an anonymous online cross-sectional survey of pregnant and postpartum women in 64 countries between May and June 2020. Participation was voluntary and no personal identifiers were collected. All potential participants were informed of the research objectives, as well as the standards of confidentiality regarding the use of the data. The survey, hosted on the Pregistry platform for COVID-19 studies (https://corona.pregistry.com), was advertised predominantly across social media platforms and online parenting forums. The advertisements and survey were made available in 12 languages (Arabic, English, French, German, Italian, Korean, Portuguese, Russian, Spanish, Simplified Chinese, Turkish, and Urdu) with translation undertaken by humans. Interested participants were invited to follow a link to take the survey. The survey collected standard demographic data and included questions that addressed topics such as COVID-19 exposure and worries, lifestyle changes, media exposure, protective factors, and mental health.

### Participants

Women who self-identified as being 18 years of age or older at the time of the survey, and as currently pregnant or having given birth within the past 6 months, were eligible to participate. The study was classified exempt by the Harvard Longwood Campus Institutional Review Board (HLC IRB) per the regulations found at 45 CFR 46.104(d) [2] on the basis that it posed no greater than minimal risk and that the recorded information was non-disclosive in nature. The total number of participants at the close of enrollment was 7561 across 64 countries.

### COVID-19 worries

Participants were asked to rate their overall level of worry about COVID-19 on a Likert Scale ranging from 1 for “not worried at all” to 4 for “very worried” [33]. They were then asked to endorse fifteen specific worries on a list developed for this study. Exploratory factor analyses were conducted to identify domains within the questionnaire with oblimin rotation on a tetrachoric correlation matrix due to the binary nature of the variables. Details of the factor analyses are presented with the supplementary material. Worries were categorized into the following domains: social (parents/grandparents unable to visit, family unable to visit, not able to have a baby shower, not able to attend a funeral), COVID-19 infection-related (participant or partner will bring infection home, family or friends will get COVID-19), child-related (no adequate childcare, other children will get COVID-19), delivery-related (partner not present during delivery, changes to delivery plan, unborn baby will get COVID-19, not able to breastfeed), economic (significantly affect economic situation/finances), and missing doctor appointments.

### Sociodemographic factors and potential confounders

Standard socio-demographic measures were collected including age, education (categorized as never attended school, elementary school, some high school, high school graduate or general equivalency diploma (GED), some college/university, college diploma or university degree, master’s degree, professional degree, doctoral degree), self-identified race/ethnicity (categorized as White/Caucasian, Latina/Hispanic, Asian, South Asian, Black, Middle Eastern, Native Hawaiian or Other Pacific Islander, American Indian or Alaska Native, Other/Multiracial), employment status (healthcare worker in a hospital or clinic, worked in a nursing home, essential/key worker, none of these, don’t know), medical coverage status, marital status (married, living with partner, divorced, separated, single, widowed), weeks pregnant/postpartum (first trimester 0 to < 13 weeks, second trimester 13 to < 28 weeks, third trimester 28+ weeks, postpartum). Participants indicated their country of residence which was classified by region for analytic purposes.

All methods carried out in this study were in accordance with relevant guidelines and regulations. All participants indicated their written conformity with a *Permission to Take Part in a Human Research Study* document. The requirement for a signed consent form was classified exempt by the Harvard Longwood Campus Institutional Review Board (HLC IRB, Protocol #: IRB20–0905) per the regulations found at 45 CFR 46.104(d) [2] on the basis that this study posed no greater than minimal risk and that the recorded information was non-disclosive in nature.

### Statistical methods

Simple tabulations and cross-tabulations were used to describe the sample (counts and percentages for categorical variables and means and standard deviation (SD) for those that were continuous). Whenever applicable, a z-test for proportions was implemented to compare responses across groups. *P*-values < 0.05 were considered statistically significant. All descriptive analyses were conducted with the programming language R [34], using the dplyr and expss packages.

Conditional logistic regression models were undertaken to determine the odds of each group of having a higher/lower worry level compared to other groups adjusting for potential confounders. Odds ratios (OR) and 95% confidence intervals (CI) were computed for each relevant variable. Worry level (1 = Not worried at all, 2 = Not very worried, 3 = Somewhat worried, 4 = Very worried) was modelled in five separate models: demographic factors, psychological constructs, media exposure variables, levels of support from family and friends, and stress levels around family and friends. All regression analyses were performed using the polr function in the R Package ‘MASS’.

## Results

### Descriptive results

We obtained a sample of at least 100 responses from each of the countries with the highest number of COVID-19 cases at the time of recruitment. In total, 7561 women participated in this study. Descriptive results are reported in Table 1.

**Table 1:** Demographic characteristics of 7561 participating pregnant and recently pregnant women

Characteristic	Percentage of participants
*Pregnancy Stage (weeks) / Postpartum*	
0–13	18.8
13–28	35.3
28–42	28.5
Postpartum	17.4
*Age Category (years)*	
< 35	72.5
> =35	27.5
*Race / Ethnicity*	
White	45.1
Latina / Hispanic	20.5
Asian	13.2
Black	8.7
Mixed Heritage	4.3
South Asian	1.8
Middle Eastern	1.3
Native	0.4
Other	3.4
*Region of Residence*	
South / Latin America	29.3
Europe	23.3
North America	17.7
Asia & Pacific	17.4
Africa	10.6
Middle East	1.8
*Marital Status*	
Married	64.8
Living with partner	26.4
Other	8.8
*Household Size*	
1	2.3
2	32.1
3	28.0
4	18.6
5 or more	19.0
*Highest Educational Attainment*	
High school graduate or less	13.8
Some college	14.4
College graduate	36.9
Graduate school or more	34.9
*Healthcare Worker / Essential Worker*	
Essential worker	13.9
Worked in healthcare or nursing home	11.3
Other	74.8
*Medical Coverage at the Time of the Pandemic*	
Yes	70.3
No	29.7

The majority (86%) of the participants reported either being “very worried” or “somewhat worried” about COVID-19 (Table 2). Women between 13 and 28 weeks of pregnancy were statistically more likely to describe themselves as “very worried” compared with the other groups—39 and 37% among those between 0 and 13 and 28–42 weeks of pregnancy, respectively, and 36% among those postpartum (*p* < 0.05). The most common source of worry, reported by 69% of participants, was that their family or friends might be infected with COVID-19. This was especially frequent among women in the postpartum stage (73% of this group reported this worry). Worrying about a partner becoming infected with COVID-19 was reported by approximately 61% of the total sample. Worrying about their unborn child being infected was reported by most respondents and especially by those in the 13 to 28 and 28 to 42 weeks pregnant cohorts (69 and 70%, respectively). Women in these categories also reported increased worry that their families would not be able to visit them after the birth and that their partners would not be able to support them during the birth (*p* < 0.05). Nearly half of the sample (45%) indicated that they had found themselves unable to stop or control worrying frequently in the previous 2 weeks (Table 3). Those who were postpartum reported this at a statistically significant lower frequency than those who were currently pregnant (*p* < 0.05).

**Table 2:** Percentage of participants and worry level and worry-related issues by pregnancy stage

How worried are you about COVID-19?	All	Pregnancy stage (weeks) / Postpartum			
		0–13	13–28	28–42	Postpartum
*Not worried at all*	2.6	3.5*	1.6*	2.0*	4.3*
*Not very worried*	11.3	11.0	10.8	12.0	11.3
*Somewhat worried*	48.4	48.3	48.3	48.7	48.3
*Very worried*	37.8	37.2	39.3*	37.3	36.0
**What about COVID-19 makes you most worried?**^a^					
*That my family members’/friends’ will be infected with COVID-19*	69.4	64.8*	69.9	69.5	73.4*
*That my partner will get COVID-19*	60.5	54.0*	62.6*	61.8	60.8
*That my family will not be able to visit me after delivery*	60.3	51.5*	64.1*	71.1*	43.6*
*That my unborn baby will get COVID-19*	60.2	58.4*	68.5*	70.2*	28.1*
*That my partner/support person will not be able to be with me during delivery because of COVID-19*	56.6	50.8*	64.9*	68.2*	25.7*
*That I will get COVID-19*	55.9	52.1*	56.1	55.1	60.9*
*That my parents/grandparents will not be able to visit the baby because of measures to stop COVID-19*	51.7	42.0*	52.7	57.4*	50.6
*That COVID-19 will mean changes to my delivery plan*	41.2	34.8*	47.1*	51.6*	18.5*
*That the COVID-19 pandemic will significantly affect my economic situation/finances*	38.9	44.6*	39.1	35.7*	37.8
*That I will not be able to have a baby shower or other baby celebration with family or friends*	35.3	31.4*	38.5*	38.1*	28.4*
*That my baby will get COVID-19*	30.8	25.3*	31.0	31.1	36.3*
*That I will be missing or cancelling doctor’s appointments*	27.2	28.1	29.4*	24.1*	27.0
*That I will not be able to breastfeed because of COVID-19*	22.4	21.6	24.5*	23.0	18.0*
*That I will not be able to provide adequate* *childcare for my other kids*	20.2	17.4*	19.5	20.4	24.2*
*That I will not be able to attend the funeral of a family member*	19.9	21.3	20.4	18.2*	20.0
**Total participants**	**7317**	**1362**	**2610**	**2101**	**1244**

*p < 0.05 comparing one cell vs all others; ^a^Respondents were asked to select all that apply

**Table 3:** Percentage of participants who answered to “How often have you been bothered by not being able to stop or control worrying during the last 2 weeks?”

	All	Percentage of participants by Pregnancy Stage (weeks) / Postpartum			
		0–13	13–28	28–42	Postpartum
*Not at all*	27.3	26.8	27.4	25.2*	31.3*
*More than half the days*	14.3	15.5	14.3	13.7	14.0
*Nearly every day*	13.9	14.0	13.3	15.0	13.0
*Several Days*	44.5	43.7	45.0	46.1	41.7*
**Total cases**	**7561**	**1418**	**2671**	**2154**	**1318**

*p < 0.05

In our conditional regression model (Table 4), we found increased age (≥35 years: OR: 1.21, 95% CI: 1.09–1.34) and being 13–28 weeks pregnant (OR: 1.15, 95% CI: 1.01–1.30) compared with 0–13 weeks pregnant to be associated with increased worry. Furthermore, those living in Africa (OR: 3.08, 95% CI: 2.59–3.66), Asia and the Pacific (OR: 1.49, 95% CI: 1.28–1.73), North America (OR: 1.27, 95% CI: 1.10–1.46), and South/Latin America (OR: 3.96, 95% CI: 3.47–4.51) experienced an increase in odds of reporting a high worry level compared with those in Europe. When the region of residence was replaced with race and ethnicity in the model, every group (other than Middle Eastern) had increased odds of reporting a high worry level compared with those who self-identified as white (Supplementary Table 2). Two factors were associated with a reduction in the odds of experiencing a high worry level: employment as an essential worker (OR: 0.84, CI: 0.73–0.95) compared with other jobs and living with a partner compared with another marital status (OR: 0.77, CI: 0.65–0.92).

**Table 4:** Conditional logistic regression of worry levels^a^ adjusted for potential confounders

Variables	Odds ratio (95% confidence interval)
*Age (years)*	
< 35	Reference
> =35	1.21 (1.09–1.34)
*Highest Education Level Attained*	
High School	Reference
Some college	1.01 (0.85–1.20)
College graduate	0.96 (0.83–1.11)
Graduate school or more	1.01 (0.87–1.17)
*Employment during the pandemic*	
Other	Reference
Essential worker	0.84 (0.73–0.95)
Healthcare or nursing home worker	0.98 (0.85–1.12)
*Marital Status*	
Other	Reference
Living with partner	0.77 (0.65–0.92)
Married	0.92 (0.77–1.09)
*Region of residence*	
Europe	Reference
Africa	3.08 (2.59–3.66)
Asia & Pacific	1.49 (1.28–1.73)
Middle East	1.18 (0.80–1.74)
North America	1.27 (1.10–1.46)
South/Latin America	3.96 (3.47–4.51)
*Household size*	1.05 (1.02–1.08)
*Medical coverage*	
No	Reference
Yes	0.93 (0.84–1.03)
*Weeks pregnant*	
0–13	Reference
13–28	1.15 (1.01–1.30)
28–42	1.06 (0.92–1.21)
Postpartum	1.12 (0.96–1.30)
*Checks COVID-19 News in traditional media per day*	
< 5 Times	Reference
≥ 5 Times	1.63 (1.33–2.00)
*Checks COVID-19 News in social media per day*	
< 5 Times	Reference
≥ 5 Times	1.78 (1.51–2.11)
*Discussed COVID-19* via *mass communication per day*	
< 5 Times	Reference
≥ 5 Times	0.87 (0.68–1.11)
*Discussed COVID-19 with others per day*	
< 5 Times	Reference
≥ 5 Times	1.62 (1.35–1.96)
*Source of support*	
Children	1.06 (1.01–1.12)
Co-workers	0.95 (0.89–1.00)
Friends	0.95 (0.88–1.02)
Husband/Partner	1.02 (0.96–1.08)
Parents	1.00 (0.94–1.07)
Siblings	1.10 (1.04–1.17)
*Source of Stress*	
Children	0.97 (0.91–1.02)
Co-workers	1.06 (1.00–1.14)
Friends	0.93 (0.85–1.01)
Husband/Partner	1.17 (1.10–1.24)
Parents	1.09 (1.02–1.16)
Siblings	1.12 (1.04–1.21)

^a^Worry Level was classified as 1 = Not worried at all, 2 = Not very worried, 3 = Somewhat worried, 4 = Very worried

In our adjusted model (Table 4), increased social media interaction was associated with increased odds of being in the highest worry category. This result was consistent across all media: checking the news ≥5 times a day (OR: 1.63, 95% CI: 1.33–2.00) and checking social media (OR: 1.78, 95% CI: 1.51–2.11). Those who discussed the news and COVID-19 frequently (≥ 5 times a day) also reported increased worry compared with those who discussed them less frequently (OR:1.62, 95% CI: 1.35–1.96).

In terms of the support that participants were able to utilize, each higher level of support from children (OR: 1.06, 95% CI: 1.01–1.12) and siblings (OR: 1.10, 95% CI: 1.04–1.17) was associated with increased odds of having the highest worry level (Table 3). No other areas of support were associated with statistically significant differences in worry level. For stress level, family stress from a husband/partner (OR: 1.17, 95% CI: 1.10–1.24), parents (OR: 1.09, 95% CI: 1.02–1.16), and siblings (OR: 1.12, 95% CI: 1.04–1.21), but not from children, were all associated with an increased odds of having a high worry level.

## Discussion

This study explored worry during the COVID-19 pandemic among an international sample of pregnant and postpartum women. We found high levels of worry in both populations. Factors such as age, region of residence, race and ethnicity, and stage of pregnancy were associated with changes in worry level. Our finding that 86% of the sample reported being either ‘somewhat’ or ‘very worried’ during the pandemic highlights the pervasive impact global crises can have on levels of worry among pregnant and postpartum women. Pandemics and their consequences (including job insecurity and economic downturn) have historically been found to increase the incidence of anxiety, depression, and worry (1). Given the impact that the mental health of parents can have upon their own future prospects (2), and the health and wellbeing of their children throughout life (3), societies should be looking at ways to further support these populations during this unusual time.

That women who are 13–28 weeks pregnant and/or aged ≥35 years of age report increased worry, compared with other women, are novel findings. The former result may be related to the specific requirements of this group, in terms of services utilization, which then increases worries related to COVID-19 infection. While the latter may be a consequence of older women being aware that increased age is associated with poor outcomes when infected with COVID-19 or related to the increased risk of pregnancy complications among this group that could require more frequent interactions with health care services. It could be hypothesized, based on our findings, that those who are most likely to need to interact with the outside world are those who report higher rates of worry.

Our data suggest significantly higher worry level among populations residing outside Europe, and in some racial and ethnic communities (Asian, Black, Latin/Hispanic, Mixed Race, South Asian and other). Several factors likely contribute to this result including: 1) differences in access and quality of health care among regions (many of the highest-ranking health care systems are found in Europe); 2) the impact being able to access health care can have upon feelings of wellbeing and health and; 3) consistently established differences in COVID-19 outcomes between racial and ethnic groups [35]. At the same time, our model found that having access to health insurance, a proxy for accessible health care, was not associated with worry level. How these overlapping, but separate, factors impact upon worry requires further investigation [36].

Seeking out information on social media was found to be significantly associated with worry level; however, we were unable to explore directionality. In other words, we could not establish whether increased worry was the cause or the consequence of heightened use of social media. Increased social media consumption and worry level during the pandemic are supported by earlier studies undertaken in the general population and among health care workers [4]. Research also reports that social media usage at the start of the pandemic had the potential to alleviate some of the negative mental health impacts of COVID-19, but that ongoing usage was detrimental [5]. This suggests that there is a cut-off point (either in terms of regularity per day or length of time) above which seeking COVID-related information online reduces, rather than improves, mental health and worry. This may be partly related to what the World Health Organization calls an “infodemic” [37]: fake news, misinformation, and conspiracy theories have skyrocketed since the beginning of the pandemic, undermining trust in health institutions and programs and increasing social anxiety and unrest. Given the high rates of social media interaction across much of the globe, governments and non-governmental organizations should consider public health campaigns to support healthy online information seeking and social media use and encourage taking breaks from being online [6].

It might be assumed that those who are pregnant or postpartum are likely to require increased support from friends and family during COVID-19. Interestingly, however, we found that higher support from children and siblings increased worry levels among our participants, and that enhanced support from other networks did not significantly reduce worries. Perhaps, the benefits of increased social networks are counterbalanced by the negative consequences of worrying about the wellbeing of loved ones or a potential for COVID-19 infection from those offering support. It is likely that individuals have been responding to the pandemic by limiting their social interactions. This might also explain why being lonelier was associated with a reduction in the odds of being in the highest category of worry in our study.

Interactions with family members can be the cause of stress and influence responses to stress across the life course [24]. Therefore, it is unsurprising that higher rates of worry among our participants are associated with greater family stress (husband/partner, parents, and siblings, but not children). Several family-related factors—including partner support, harmonious family environment, parenting style, and having parents with mental health problems—have all been found to alter an individual’s anxiety and stress during the COVID-19 pandemic [26]. The effects of population interventions, such as quarantines and lockdowns, on family dynamics are not yet well understood and this is an ongoing area of investigation. The significant associations between psychological constructs and increased levels of worry may also be determined by the social networks and interactions individuals have.

It is noteworthy that those who identified themselves as essential workers had lower odds of being in the highest worry category. This is contrary to previous research which has found that those working at the front-line, including health care workers, are likely to experience increased rates of anxiety compared with the general public [27]. Women in these roles also appear to be at an increased risk of experiencing mental health disturbances as a result of COVID-19 [28]. The reasons for these discrepancies are unclear and need to be investigated further.

Our study is limited to the perspectives and experiences of a selected group of pregnant and postpartum women. While this is an international study, we were unable to compare countries because of an over-representation of respondents from the United States, Mexico, and South Africa, with a long tail of fewer responses from many other countries. Over-representation of countries is a result of our survey distribution methods. Another limitation of the study is that the sample size is not consistent across all analyses. This is due to missing responses (i.e. participants that did not answer every question) and re-coding of data to only included responses of ‘Yes’ or ‘No’ for the chi-square analyses. Study findings are limited to the point in time and phase of the pandemic when data were collected (May and June 2020) and should be interpreted as a snapshot of a rapidly changing global pandemic. Further, as a result of the cross-sectional study design, it was not appropriate to statistically examine whether and how people and countries felt as compared to others preceding them. Despite these limitations, the present study clearly shows that the pandemic and the restrictions imposed on the population have greatly impacted prospective mothers’ and postpartum women’s level of worry, placing their mental health and emotional stability at stake.

How postpartum depression might add to the worry and stress caused by COVID-19 is poorly understood. Given that approximately one in seven women develop postpartum depression [38], and that the societal and individual consequences of COVID-19 affect the mental health of those who are pregnant more than of those who are not pregnant [39], supporting this cohort during the pandemic should be considered an important public health issue. Further research is much needed to determine how best to provide such support.

## Conclusions

Despite COVID-19 being a novel pandemic, there is a growing body of evidence that suggests an already significant impact on the psychological wellbeing of the general population [29]. The results presented here indicate increased levels of worry among pregnant and postpartum women during this time, which highlights the importance of imminent mental health challenges in these populations that may further increase as COVID-19 continues. Data are beginning to emerge showing that those most vulnerable to changes to health care provision and the economy due to the pandemic are more likely to experience significantly increased worry and a negative effect on their wellbeing [30]. As a result, there is an urgent need for prevention and wellbeing services. Understanding the needs of these populations during the unfolding pandemic is essential to their health and the health of future generations.

## Supplementary Information

**Additional file 1.** IRCEP-HSPH Wellbeing Survey.pdf Health and Wellbeing of Pregnant and Post-Partum Women During the COVID-19 Pandemic Questionnaire. Survey used to assess the mental health and wellbeing of pregnant and recently pregnant women during the COVID-19 pandemic in 64 countries

## Data Availability

The datasets used and/or analyzed during the current study are available from the corresponding author on reasonable request.

## Abbreviations

CI: Confidence intervals
COVID-19: Coronavirus disease
GED: General equivalency diploma
HLC IRB: Harvard Longwood Campus Institutional Review Board
OR: Odds ratios
SD: Standard deviation
