# Supplementary material for: Frequency and source of worries in an International sample of pregnant and postpartum women during the Covid-19 pandemic
Source: BMC Pregnancy Childbirth. 2021 Nov 12;21:768. doi: 10.1186/s12884-021-04241-2 (PMC8586614; doi:10.1186/s12884-021-04241-2)
Supplement: Supplementary file 1 — Additional file 1. IRCEP-HSPH Wellbeing Survey.pdf Health and Wellbeing of Pregnant and Post-Partum Women During the COVID-19 Pandemic Questionnaire. Survey used to assess the mental health and wellbeing of pregnant and recently pregnant women during the COVID-19 pandemic in 64 countries [file 12884_2021_4241_MOESM1_ESM.pdf]

Frequency and Source of Worries in an International Sample of Pregnant and Postpartum Women During the COVID-19 Pandemic

Diego F. Wyszynski, Sonia Hernandez-Diaz, Vanessa Gordon-Dseagu, Noemi Ramiro, Archana Basu, Hannah H. Kim, Karestan C. Koenen

Health and Wellbeing of Pregnant and Post-Partum Women During the COVID-19 Pandemic

Questionnaire

A collaboration of the Harvard T.H. Chan School of Public Health and Pregistry

Principal Investigator: Karestan C. Koenen, PhD (kkoenen@hsph.harvard.edu)
Co-Investigator: Sonia Hernandez-Diaz, MD, DrPh (shernan@hsph.harvard.edu)
Co-Investigator: Diego Wyszynski, MD, MHS, PhD (diegow@pregistry.com)

This document is the property of Pregistry, LLC. It is intended for restricted use only and may not – in full or part – be passed on, reproduced, published or used without express permission of Pregistry, LLC. Copyright © 2020 by Pregistry, LLC. All rights reserved.

Table of Contents

Demographics 3
COVID-19 Questions........ 5
COVID-19 & Media Exposure 6
Impact of COVID-19 6
Support system 11
Recent Experiences........ 12
Feedback 13

Demographics

In which country do you live? *
(Dropdown) options: list of country.

In which state/province do you live?
(Text Field)

In which city do you live?
(Text Field)

Are you pregnant? *
- Yes
- No

(Radio Button)

[If selects "yes"]
How many weeks pregnant are you? *
(Dropdown) options: list from 5 to 43.

[If selects "No"]
How long ago did you give birth? *
(Dropdown) options: Less than one month ago, One month ago, Two months ago, Three months ago, Four months ago and Five months ago.

How old are you? *
(Dropdown) options: list from 18 to 50.

Which race(s) do you most identify with? (check all that apply)
- White/Caucasian
- Latin/Hispanic
- Asian
- South Asian
- Black
- Middle Eastern
- Native Hawaiian or Other Pacific Islander
- American Indian or Alaska Native
- Other/Multirace (Text Field)

(Checkbox)

What is your marital status?

- Single
- Married
- Living with partner
- Separated
- Divorced
- Widowed

(Radio Button)

How many people live in your household (including yourself)?

(Dropdown) options: list from 1 to 10 and More than 10.

What is the highest level of education you attained?

- Never attended school
- Elementary school
- Some high school
- High school graduate or general equivalency diploma (GED)
- Some college/university
- College diploma or university degree
- Masters degree
- Professional degree
- Doctoral degree.

(Radio Button)

Please indicate if during the COVID-19 pandemic you:

- Were a healthcare worker in a hospital or clinic
- Worked in a nursing home
- Were an essential/key worker (as defined by the government)
- None of these
- Don’t know

(Radio Button)

Do you have medical insurance through your government or your employer?

(Dropdown) options: Yes and No.

COVID-19 Questions

Have you been tested for SARS-CoV-2 (the virus that causes COVID-19)? If so, what was the result?

- Positive, I had the virus
- Negative, I did not have the virus
- Yes, but I do not know the result yet or the result was inconclusive
- No, I have not been tested.

(Radio Button)

Has a health care professional (e.g., doctor, nurse) diagnosed you as having COVID-19 based on your symptoms only?
(Dropdown) options: No, Yes, and I still have it, Yes, but I recovered.

Have you been in contact with an individual who has or had COVID-19?
(Dropdown) options: Yes, No and Maybe.

Which of the following have you done in the last 7 days to keep yourself and others safe from COVID-19? (Select all that apply)

- Wore a face mask
- Washed your hands with soap or used hand sanitizer several times per day
- Disinfected surfaces around you
- Stockpiled hand sanitizer or disinfectant wipes
- Stockpiled food or water
- Canceled or postponed air travel for work
- Canceled or postponed air travel for pleasure
- Canceled or postponed work or school activities
- Canceled or postponed personal or social activities
- Avoided contact with people who could be high-risk
- Avoided public spaces, gatherings, or crowds
- Avoided eating at restaurants
- Worked or studied at home
- Visited a doctor
- Canceled a doctor’s appointment
- Stockpiled medication
- Prayed

(Checkbox)

COVID-19 & Media Exposure

	Never	<1 x/day	1 x/day	2-4 x/day	5-8 x/day	9-16 x/day	>16 x/day
**How often do you check the news about COVID-19?**	●	●	●	●	●	●	●
**How often do you check social media about COVID-19? (e.g. WhatsApp, Facebook)**	●	●	●	●	●	●	●
**How often do you discuss COVID-19 in mass communications? (e.g. WhatsApp group, Twitter)**	●	●	●	●	●	●	●
**How often do you discuss COVID-19 with another person?**	●	●	●	●	●	●	●

(Radio Button)

Impact of COVID-19

How worried are you about COVID-19? *
(Dropdown) options: Very worried, Somewhat worried, Not very worried, Not worried at all. (skip next question).

[If selects "Not worried at all"]
(skip next question)
[If selects anyone from these "Very worried, Somewhat worried, Not very worried"]

What about COVID-19 makes you most worried? Select all that apply: *

- That I will get COVID-19
- That my partner will get COVID-19
- That my family members'/friends' will be infected with COVID-19
- That the COVID-19 pandemic will significantly affect my economic situation/finances

Confidential – ©Pregistry, LLC – 2020

- That my unborn baby will get COVID-19
- That COVID-19 will mean changes to my delivery plan
- That my partner/support person will not be able to be with me during delivery because of COVID-19
- That my family will not be able to visit me after delivery
- That my baby will get COVID-19
- That my parents/grandparents will not be able to visit the baby because of measures to stop COVID-19
- That I will not be able to breastfeed because of COVID-19
- Other (Text field)

(Checkbox)

To what extent has COVID-19 negatively impacted the following areas of your life? *

	Not at all	A little bit	Moderately	A lot
**Sleep**	●	●	●	●
**Diet**	●	●	●	●
**Fitness**	●	●	●	●
**Work**	●	●	●	●
**Finances**	●	●	●	●
**Family**	●	●	●	●
**Relationships**	●	●	●	●
**Religious observance**	●	●	●	●
**Character (e.g., patience, trust)**	●	●	●	●

(Radio Button)

Since the COVID-19 pandemic started, how often have any of the following things happened to you?

	Almost every day	At least once a week	A few times a month	Never
**You were treated with less courtesy than other people are**	●	●	●	●
**You received poorer service than other people at restaurants or stores**	●	●	●	●
**People acted as if they are afraid of you**	●	●	●	●
**You were called names or insulted**	●	●	●	●
**You were threatened or harassed**	●	●	●	●
**You were physically assaulted**	●	●	●	●

(Radio Button)

[If selects "Never" for all]
(skip next question)

[If selects any of the options except "Never"]
What do you think is the main reason for these experiences? (Choose all that apply)

- Your ancestry or national origins
- Your gender
- Your race or ethnicity
- Your age
- Your religion
- Your height
- Your weight
- Your pregnancy
- Your sexual orientation
- Your education or income level
- Other (Text field)

(Checkbox)

Please answer the following questions based on the last 2 weeks.

	Never	Almost Never	Sometimes	Fairly Often	Very Often
How often have you felt that you were unable to control the important things in your life?	•	•	•	•	•
How often have you felt confident about your ability to handle your personal problems?	•	•	•	•	•
How often have you felt that things were going your way?	•	•	•	•	•
How often have you felt difficulties were piling up so high that you could not overcome them?	•	•	•	•	•

(Radio Button)

How often have you been bothered by the following problems during the last 2 weeks? *

	Not at all	Several Days	More than half the days	Nearly every day
Feeling nervous, anxious or on edge	•	•	•	•
Not being able to stop or control worrying	•	•	•	•
Feeling down, depressed or hopeless	•	•	•	•
Little interest or pleasure in doing things	•	•	•	•

(Radio Button)

Since the COVID-19 crisis began, how often do you eat...

	Almost never or never	Rarely	Sometimes	Often	Almost always or always
Because you're depressed or sad	●	●	●	●	●
As a way to help you cope	●	●	●	●	●
As a way to comfort yourself	●	●	●	●	●
Because you feel worthless or inadequate	●	●	●	●	●
As a way to avoid thinking about something unpleasant or to distract yourself	●	●	●	●	●

(Radio Button)

Indicate how much you agree with the following statements *

	Strongly disagree	Disagree	Somewhat disagree	Neither agree nor disagree	Somewhat agree	Agree	Strongly agree
I have so much in life to be thankful for	●	●	●	●	●	●	●
If I had to list everything that I felt grateful for, it would be a very long list	●	●	●	●	●	●	●
I am grateful to a wide variety of people	●	●	●	●	●	●	●

(Radio Button)

Please describe how the COVID-19 crisis has impacted you the most:
(Text Field)

Support system

Since the COVID-19 crisis began, how supportive are the following people?

	Not at all	A little bit	Moderately	Quite a bit	Extremely	N/A (there is no such person)
Husband or significant other	●	●	●	●	●	●
Parents or legal guardians	●	●	●	●	●	●
Children	●	●	●	●	●	●
Siblings	●	●	●	●	●	●
Friends	●	●	●	●	●	●
Co-workers	●	●	●	●	●	●

(Radio Button)

Since the COVID-19 crisis began, how stressed do you feel by the following people?

	Not at all	A little bit	Moderately	Quite a bit	Extremely	N/A (there is no such person)
Husband or significant other	●	●	●	●	●	●
Parents or legal guardians	●	●	●	●	●	●
Children	●	●	●	●	●	●
Siblings	●	●	●	●	●	●
Friends	●	●	●	●	●	●
Co-workers	●	●	●	●	●	●

(Radio Button)

Since the COVID-19 crisis began… *

	Hardly ever	Some of the time	Often
How often do you feel that you lack companionship?	●	●	●
How often do you feel left out?	●	●	●
How often do you feel isolated from others?	●	●	●

(Radio Button)

Confidential – ©Pregistry, LLC – 2020

Recent Experiences

Thinking about your experience IN THE PAST 7 DAYS, please indicate how strongly you agree or disagree with each of the following statements

	Strongly agree	Agree	Somewhat agree	Neither agree nor disagree	Somewhat disagree	Disagree	Strongly disagree
I don’t feel I belong to anything I’d call a community	●	●	●	●	●	●	●
I have something valuable to give the world	●	●	●	●	●	●	●
I feel close to other people in my community	●	●	●	●	●	●	●
I cannot make sense of what’s going on in the world	●	●	●	●	●	●	●
My community is a source of comfort	●	●	●	●	●	●	●
I believe that people are kind	●	●	●	●	●	●	●

(Radio Button)

Please answer the following questions based on the last 7 days

	Not at all	A little bit	Moderately	Quite a bit	Extremely
I feel watchful or on-guard	●	●	●	●	●
Other things keep making me think about COVID-19	●	●	●	●	●
I am aware that I have a lot of feelings about COVID-19, but I don't deal with them	●	●	●	●	●
I try not to think about COVID-19	●	●	●	●	●
I have trouble concentrating	●	●	●	●	●

(Radio Button)

Feedback

We would love to receive your feedback about this survey:

(Text Field)
